# Switching Roles of TGF-β in Cancer Development: Implications for Therapeutic Target and Biomarker Studies

**DOI:** 10.3390/jcm5120109

**Published:** 2016-11-30

**Authors:** Nan Sun, Ayumu Taguchi, Samir Hanash

**Affiliations:** 1 Department of Clinical Cancer Prevention, the University of Texas MD Anderson Cancer Center, Houston, TX 77030, USA; nsun@mdanderson.org; 2 Department of Translational Molecular Pathology, the University of Texas MD Anderson Cancer Center, Houston, TX 77030, USA; ataguchi@mdanderson.org

**Keywords:** TGF-β, targeted therapy, role switch, biomarker

## Abstract

TGF-β induces complicated and even opposite responses in numerous biological processes, e.g., tumor suppression in pre-malignant cells and metastasis promotion in cancer cells. However, the cellular contextual determinants of these different TGF-β roles remain elusive, and the driver genes triggering the determinants’ changes have not been identified. Recently, however, several findings have provided new insights on the contextual determinants of Smads in TGF-β’s biological processes. These novel switches and their effectors may serve as prognostic biomarkers and therapeutic targets of TGF-β-mediated cancer progression.

## 1. Introduction

Transforming growth factor-β (TGF-β) plays key roles in many biological functions, such as embryonic stem cell self-renewal and differentiation, homeostasis of differentiated cells, suppression of the immune system, and promotion of cancer development [1]. The TGF-β signaling pathway has been well characterized. TGF-β binds to its receptor on the cell membrane and induces a signaling cascade by phosphorylating Smad2/3. Phosphorylated Smad2/3 binds to Smad4, and the complex translocates from the cytoplasm to the nucleus to activate the transcription of end effectors such as p15, p21, and PTHrP [2,3]. In normal epithelial cells, TGF-β induces the activation of cytostatic genes, including *p15* [4] and *p21* [5,6,7]. TGF-β also inhibits a set of genes that promote cell growth, including *c-MYC* [8,9,10]. In addition to the canonical TGF-β/Smad signaling pathway, TGF-β can activate several non-canonical signaling pathways. For example, TGF-β regulates Erk, p38, MAPK, JNK, PI3K-Akt, and small GTPases [11,12]. 

An emerging notion is that cellular contextual functionality, more than TGF-β itself, dictates the complicated and even opposite natures of TGF-β-induced responses. In this review, we summarize recent findings about the role switching of TGF-β in cancer development; these findings shed light on potential therapeutic targets in the TGF-β signaling pathway and predictive biomarkers for anti-TGF-β therapy.

## 2. The Context-Dependent Functions of TGF-β in Normal Tissues

Although the signaling cascade of TGF-β involves only a few Smad proteins and seems simpler than other receptor-mediated signaling pathways, the cellular responses to TGF-β are complicated and are highly dependent upon the cellular context [1]. Most of this context dependency can be explained by the interactions between Smads and a wide-ranging complement of DNA sequence-binding transcription factors, including p53 and members of the bHLH, Forkhead box (Foxo), and Zinc finger protein families [13]. For example, in neuroepithelial and glioblastoma cells, TGF-β induces Smad3/4 to form a complex with Foxo3a to activate *p21* gene expression [5]. However, several other reports showed that this complex does not exist in human mammary epithelial cells. Instead, an alternative mechanism for p21 activation in response to TGF-β, involving p53 and Smad2/3/4, has been identified in a mammary epithelial cell line MCF-10A [14,15,16,17]. Interestingly, TGF-β reportedly induces *p21* through a p53-independent mechanism in the HaCaT cell line, which contains two mutant alleles of p53 that cannot activate transcription of *p21* [18]. Taken together, these data show that TGF-β’s signaling program is highly dependent on cell context.

It has been known for decades that TGF-β induces complicated and even opposite responses in many biological processes as a consequence of various contextual determinants for Smad. In particular, Smads activate a lineage-specific transcriptional program in cooperation with lineage-specific transcription factors during cell differentiation [19,20]. For example, TGF-β–activated or bone morphogenetic protein–activated Smads collaborate with MYOD1 in myoblasts or with PU.1 in pre-B cells [19]. Additionally, CCAAT-enhancer binding protein (C/EBP)–α recruits Smads in myeloid precursors, while GATA1 works with Smads in erythroid precursors to initiate differentiation into these two lineages [20]. In embryonic stem cells, the same Smad complexes bind to FOXH1, a mesendoderm lineage factor, to initiate the expression of multiple differentiation genes [21,22]. However, bone morphogenetic protein-activated Smad1 and the leukemia inhibitory factor mediator STAT3 form a self-renewal network with the pluripotency core complex OCT4-SOX2-NANOG in embryonic stem cells [23,24]. Moreover, TGF-β plays a role in regulating the expression of normal tissue homeostasis genes such as *SERPINE1*, *CDKN1A*, *MYC*, and *ID1* in differentiated cells with various Smad co-transcription factors, including AP1, FOXO1, E2F4, and ATF3, respectively [5,25,26,27,28,29]. 

## 3. The Context-Dependent Functions of TGF-β in Cancer Development

TGF-β is primarily a tumor suppressor that inhibits proliferation or induces apoptosis of premalignant epithelial cells [29]. In the later stages of cancer progression, however, TGF-β functions as a metastasis promoter by inducing epithelial-mesenchymal transition (EMT), leading to increased invasion of cancer cells, and by inducing genes that facilitate metastatic colonization of secondary organ sites (e.g., lung, bone, liver, and brain) [29]. Although the opposing functions of TGF-β in early- and late-stage cancer have been known for years, it is unclear how and when TGF-β switches from tumor suppressor to metastasis promoter. An emerging notion is that cellular contextual functionality dictates the divergent roles of TGF-β. However, the cellular contextual determinants for Smads in response to TGF-β in different cells and the driver genes that trigger the changes of contextual determinants for Smads have never been well understood.

Several studies have attempted to identify these determinants and drivers. For example, TMEPAI knockdown attenuates TGF-β-induced growth and motility in breast cancer cells [30]. miR-106b was identified as a molecular switch that determines TGF-β’s effects on cell proliferation, is elevated in late-stage tumors, and correlates with tumor progression in breast cancer patients. TGF-β increases the transcription of miR-106b by activating c-Jun to bind to its promoter. However, miR-106b upregulation counterbalances the growth-inhibiting effects by abolishing activated retinoblastoma protein, resulting in enhanced proliferation. Thus, as a downstream target of TGF-β, miR-106b could direct the tumor-promoting functions of TGF-β in breast cancer [31]. In an established model of TGF-β-induced EMT in mouse mammary gland epithelial cells, C/EBPβ is repressed by miR-155, an oncomiR. Depletion of *C/EBPβ* enhances TGF-β-induced EMT by decreasing the transcription of E-cadherin and of the coxsackie virus and adenovirus receptor, contributes to evasion of the growth-inhibitory effect of TGF-β, and further enhances invasion and metastatic dissemination of the mouse mammary tumor cells to the lungs after subcutaneous injection into mice [32]. The role switching of TGF-β from tumor promoter to tumor suppressor is also shown in the reprogramming of MDA-MB-231 triple-negative breast cancer cells with the *GATA3* transcription factor. *GATA3* overexpression in these cells reduces TGF-β response, reverses EMT, and restores sensitivity to TGF-β’s inhibitory effects on cell proliferation in MDA-MB-231 cells. In addition, overexpression of *GATA3* in MDA-MB-231 cells results in the reprogramming of these cells from a basal to a luminal subtype, which has been associated with reduced metastasis and reduced tumorigenesis in a xenograft model [12]. Latency-associated protein, an active isoform of transcription factor C/EBPβ, is essential for TGF-β induction of the cell cycle inhibitor p15INK4b together with a Foxo-Smad complex and repression of c-MYC with an E2F4/5-Smad complex in human mammary epithelial cells. However, the cytostatic response is selectively missing in metastatic cancer cells and patient samples owing to an excessive expression of LIP, which is the natural dominant negative inhibitory isoform of C/EBPβ. These data suggest that C/EBPβ, especially the ratio of LIP to latency-associated protein, plays a key role in the coordination of TGF-β cytostatic responses, and its malfunction may trigger evasion of these responses in cancer [25].

It has been suggested that mutated *p53* switches TGF-β from a tumor suppressor to a metastasis promoter. *p53* is the most frequently mutated gene as cancer evolves [33] and has been identified as a Smad binding partner for TGF-β-induced *p21* gene expression and cytostatic function [14,16,17,34,35]. Wild-type p53 inhibits TGFβ-induced EMT and EMT-associated stemness in a mammary epithelial cell model [36,37]. A possible mechanism of this inhibition is that p53 regulates the expression of EMT mediator-*Zeb1/2* and stem cell executor-*BMI1* through transcriptional activation of miR-200c [37], which is also a downstream target of the TGF-β signaling pathway [38,39,40]. Meanwhile, expression of mutant p53 (R273H) or loss of p53 inhibits the suppressive function of TGF-β in cell proliferation in an ovarian cancer cell line [41]. An important study which was conducted in lung cancer cell line H1299 and ovarian cancer cell line SKOV3 showed that cells expressing mutant p53 lost their sensitivity to TGF-β. This is because the mutant p53 attenuates TGF-β signaling and TGF-β-induced transcription activity of Smad2/3 proteins by reducing the expression of TGF-β type II receptor [42]. These studies provide insight into the molecular mechanisms of mutant p53 “gain of function” pertaining to the TGF-β signaling pathway.

TGF-β plays a range of roles in various types of cancer. In prostate tumorigenesis, *PPARδ* is a direct transcription target of TGF-β and plays a critical role in switching the function of TGF-β. *PPARδ* induction inhibited TGF-β-mediated growth inhibition, while its activation promoted TGF-β-induced tumor growth, migration, and invasion. Mechanistically, TGF-β activation of the PPARδ-ABCA1-Cav1 pathway facilitates degradation of TGF-β receptors and attenuates Smad activity and growth inhibitory function but enhances the ERK signaling pathway in response to TGF-β [43].

Changes in the cellular microenvironment including tissue stiffness and matrix rigidity could also affect the functional response to TGF-β. Low rigidity increases TGF-β-induced apoptosis, but high rigidity results in EMT. Matrix rigidity does not change Smad signaling; instead it regulates the PI3K/Akt signaling pathway, which also plays a critical role in the apoptotic and EMT responses. These findings provide insight into how tissue mechanics might contribute to the cellular response to TGF-β [44].

The above studies have partially revealed TGF-β’s functions and role switch in cancer development. However, not all of these studies exactly explain how TGF-β loses its tumor suppressor function during cancer initiation, as most of the data were obtained from cancer cell lines in which TGF-β had already gained a tumor promoter function. Moreover, most of the TGF-β studies were focused on cell proliferation and tumor growth, which are only part of TGF-β’s functions. More effort is needed to comprehensively understand the mechanism of the role switch of TGF-β. 

## 4. Challenges in Current Cancer Therapy Targeting TGF-β and Implications for Biomarker Studies

Since the TGF-β signaling pathway plays an important role in cancer development and a variety of other diseases, much effort has gone toward developing cancer therapeutics to target TGF-β signaling in both the tumor and its microenvironment [45,46,47,48,49]. Currently, several TGF-β signaling antagonists, including antisense molecules, ligand traps for inhibition of the ligand-receptor interaction, anti-receptor monoclonal antibodies, TGF-β receptor kinases inhibitors, and aptamers, have been developed and applied to clinical practice [50]. 

The critical function of the TGF-β pathway in cancer, especially in the context of metastasis, spurred the development of TGF-β antagonists, yet these showed limited clinical efficacy [51,52]. In limited phase I and II trials for treating cancers generally overexpressing TGF-β, some patients with advanced cancers received a marginal benefit from TGF-β inhibitors [53,54]. However, more antagonists were developed and passed the pre-clinical trials, and they are now tested in ongoing Phase I/II clinical trials [55]. Considering the opposing roles of TGF-β in cancer, it is not surprising that its general inhibition may have unexpected deleterious consequences. Inhibiting TGF-β may accelerate the development of pre-neoplastic lesions in which TGF-β still acts as a tumor suppressor. For example, conditional knockout of *TGFBR2* in the mammary gland before tumors were established resulted in shorter median tumor latency and more pulmonary metastases [56]. Moreover, attenuated TGF-β signaling along with high *VEGFA* expression was correlated with shorter distant metastasis-free survival in HER2+ breast cancer patients [57]. In contrast, a short induction of TGF-β expression after tumors were established clearly accelerated metastatic progression [58].

**Figure 1:**
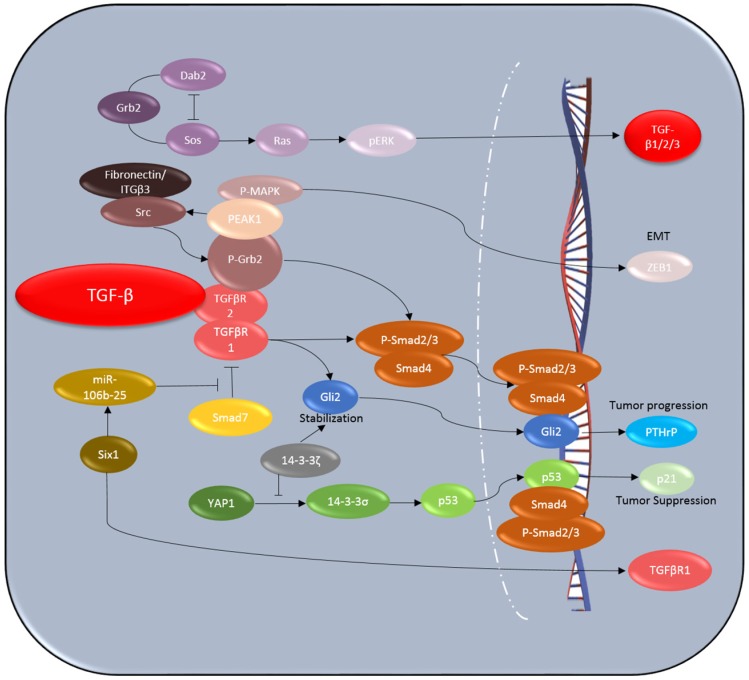
Schematic diagram of the context-dependent TGF-β signaling pathway.

As tumors evolve, TGF-β switches its role from tumor suppressor to tumor promoter. The complex nature and dual roles of TGF-β in cancer have impeded the development of effective therapies that target only the tumor-promoting activities of TGF-β. Thus, screening the individual genetic background as well as the tumor microenvironment will be highly beneficial for predicting patients’ responses to a TGF-β antagonist. The key players of the TGF-β signaling pathway are previous biomarkers such as TGFβR2, Smad2, and Smad4 by tumor biopsy or genetic analysis [59,60,61,62,63]. However, several reports showed that loss of *TGFβR2* expression is associated with more aggressive tumor behavior and reduced survival in human lung adenocarcinoma and squamous cell carcinoma [64]. These contradicting reports suggest that these key players of the TGF-β signaling pathway are not always reliable for predicting response to TGF-β inhibitory therapy and may have totally opposite, misleading impacts. Significant advances have been made in understanding the molecular mechanisms by which TGF-β switches from tumor suppressor to promoter, and several proteins that are changed during the switch, such as Six1, Dab2, 14-3-3ζ, PEAK1, p53, and Gli2, could serve as new biomarkers (Figure 1). Meanwhile, it is essential to develop circulating biomarkers to identify patients who are sensitive to TGF-β-targeting therapy and to determine the timing of patients’ responses to TGF-β antagonists to improve therapeutic efficacy. There could be additional noninvasive, blood-based biomarkers for predicting individual patient response to TGF-β inhibitors. Since TGF-β inhibitors may modulate the immune system, circulating autoantibodies could be used to monitor response [52]. Any of the following biomarkers alone or in combination could be used in the future to predict tumor response to TGF-β inhibition.

### 4.1. Six1

Six1 is a developmentally regulated homeoprotein that has frequent misexpression in various types of cancer but shows limited expression in most normal adult tissues. Overexpression of human *Six1* in adult mouse mammary gland epithelium induces aggressive mammary tumor formation and EMT in a dose-dependent manner [65]. Six1 is correlated with nuclear Smad3 and increases TGF-β signaling, promoting metastasis and relapse in breast cancer [66]. Furthermore, the same group showed more evidence that Six1-induced upregulation of TGF-β type I receptor is required to switch TGF-β signaling to the prometastatic phenotype [67]. They also identified another mechanism by which Six1 upregulates the miR-106b-25 microRNA cluster to target inhibitory Smad7, resulting in increased levels of TGF-β type I receptor and activation of TGF-β signaling later [11]. A similar function in which Six1 coordinates with TGF-β signaling and promotes EMT was also observed in cervical cancer [68]. Together these findings suggest that Six1 can switch the role of TGF-β to tumor promoter, and it could serve as a therapeutic target and prognostic biomarker.

### 4.2. Dab2

Disabled-2 (Dab2), a structural homologue of the Dab1 adaptor molecule, acts as a critical link between the TGF-β receptors and Smads [69]. Previous studies showed that TGF-β-induced *Dab2* expression levels block visceral endoderm differentiation, stimulate JNK activity, and promote cell migration [70,71]. Moreover, via transcript-selective translational induction of *Dab2*, TGF-β-mediated phosphorylation of hnRNP E1 can induce EMT [72]. However, recent studies have demonstrated that the downregulation of *Dab2* expression via promoter methylation is an independent predictor of metastasis and poor prognosis in squamous carcinoma [73]. Downregulation of *Dab2* abrogates the TGF-β tumor suppressor function by blocking TGF-β-mediated inhibition of cell proliferation and migration and facilitates TGF-β-stimulated EMT [74]. A study in which Dab2 was re-expressed in SK-BR-3 cells found that TGF-β was depleted in the surrounding medium via normalization of the trafficking of TGF-β receptors [75]. Moreover, low expression of Dab2 occurred in esophageal squamous cell carcinoma, associated with poor survival and high recurrence [76]. Together, these data indicate that Dab2 is an important regulator of TGF-β signaling, which could aid in the inhibition of cancer and the selection of patients for anti-TGF-β therapies.

### 4.3. 14-3-3*ζ*

Studies have shown that *14-3-3*ζ overexpression occurs in early-stage breast cancer (atypical ductal hyperplasia) [77] and has a high correlation with recurrence [78]. *14-3-3*ζ overexpression activated the TGF-β/Smad pathway, which led to ZFHX1B/SIP-1 upregulation, E-cadherin loss, and EMT [79]. TGF-β induces *p21* expression and cytostatic function in non-malignant HMECs through the p53/Smad complex. Surprisingly, a recent study found direct evidence that overexpression of *14-3-3*ζ inhibits TGF-β’s cell cytostatic program in non-transformed human mammary epithelial cells, while overexpression of *14-3-3*ζ promotes TGF-β-induced metastatic colonization of bone by breast cancer. *14-3-3*ζ overexpression reduces p53, a determinant for Smads in pre-malignant cells, and thus disrupted the p53/Smad complex and inhibited TGF-β’s cytostatic function. Conversely, 14-3-3ζ stabilizes Gli2, a determinant for Smads in late-stage cancer, forming a complex with Smads to promote TGF-β-induced bone metastasis of breast cancer. Together, these results identify 14-3-3ζ as a novel molecular switch of TGF-β’s function by alteration of contextual determinants for Smads, from p53 in pre-malignant cells to Gli2 in late-stage cancers [80]. This finding suggests that 14-3-3ζ can switch TGF-β from tumor suppressor to tumor promoter, indicating that it could be a novel biomarker to aid in the selection of the patients who and when will benefit from TGF-β antagonists [29,51].

### 4.4. PEAK1

Pseudopodium-enriched atypical kinase 1 (PEAK1), Sgk269, is a 190 kDa non-receptor tyrosine kinase that controls cell spreading, migration, and proliferation [81,82]. Amplified *PEAK1* levels were found in colon cancer, pancreatic cancer, and breast cancer, suggesting that it is a potential therapeutic target [82,83]. However, a study in gastric cancer showed 71.1% negative expression of PEAK1 in the cancer tissues and indicated that loss of PEAK1 may activate EMT and promote cancer development [84]. Recently, studies indicated that PEAK1 acts as a molecular switch that regulates context-dependent TGF-β responses in breast cancer [85]. High expression levels of PEAK1 cause the loss of the anti-proliferative effects of TGF-β and initiate TGF-β-induced proliferation, EMT, cell migration, and tumor metastasis with the presence of fibronectin by switching TGF-β signaling from the canonical Smad2/3 pathway to Src and MAPK signaling. Moreover, PEAK1 is necessary for TGF-β-induced ZEB1-mediated EMT in the presence of fibronectin/ITGB3 activation [86]. Thus, PEAK1 can be used to determine when TGF-β blockade is viable in targeted therapy of breast cancer.

### 4.5. p53

Since p53 has been reported to be a critical Smad partner and to be responsible for TGF-β’s cytostatic function, p53 can be used as a biomarker for selecting patients whose TGF-β has lost its inhibitory effect on cell proliferation. In the late stages of cancer development, *p53* is lost or mutated in approximately 50% of cases [87]. In addition, p53 mutation could contribute to TGF-β’s function switch. As shown in a previous study, an R280K mutation was found in p53 in MDA-MB-231 cells, and this mutant p53 can still form a complex with Smads and enhance TGF-β-induced metastasis [15]. Furthermore, loss of p53 has been reported to induce EMT in HMECs [88], suggesting that p53 loss occurring downstream of 14-3-3ζ may be a switching point in the role of TGF-β, similar to p53 mutation [15]. Recently, a study in lung cancer showed that the radiosensitizing effect of inhibition of TGF-β signaling by SB431542 in non-small cell lung cancer cells was p53-dependent, also suggesting that p53 should be considered during anti-TGF-β treatment [89].

### 4.6. Gli2

*Gli2* is overexpressed in a variety of cancers and has a direct function in cell cycle progression, apoptosis, invasion, and metastasis [90,91]. It has been reported that TGF-β treatment induced *Gli2* mRNA transcription in MDA-MB-231 cells [92]. Additionally, studies have shown that TGF-β induced an immediate increase in Gli2 protein via 14-3-3ζ-mediated stabilization of Gli2 in MDA-MB-231 cells, suggesting that TGF-β induces Gli2 expression at multiple levels, not simply by transcriptional upregulation. TGF-β is known to induce parathyroid hormone-related protein (*PTHrP*) expression via Gli2, independently of the canonical Hedgehog pathway, enhancing bone metastasis [93]. It was reported that TGF-β-activated Smads transcriptionally unregulated Gli2, which subsequently transcriptionally unregulated *PTHrP* [92]. Recently, it was found that TGF-β-activated Smads can directly bind to Gli2 protein stabilized by 14-3-3ζ and that the Smad/Gli2 complex transcriptionally upregulates *PTHrP* [80]. This indicates that TGF-β-activated Smads can induce *PTHrP* expression via Gli2 by at least two mechanisms: (a) indirectly, via Gli2-induced *PTHrP* transcription; and (b) directly, by forming a complex with Gli2 to turn on *PTHrP* transcription. These findings suggest that Gli2 could serve as a biomarker for monitoring the role switch of TGF-β.

### 4.7. Circulating TGF-β and TGF-β-Associated Markers

The most direct biomarkers for patient selection for anti-TGF-β antagonists are circulating TGF-β in blood [94] and p-Smad2 levels in peripheral mononuclear cells [95]. Circulating TGF-β was assessed in previous studies in the blood samples of breast cancer patients [96]. The level of TGF-β in advanced-stage breast cancer was much higher than in early-stage breast cancer and has been associated with poor prognosis [97]. Another study, of an autoimmune disease, showed that the level of circulating TGF-β, which could help suppress immune functions, was elevated while the level of autoantibodies was decreased in blood [98]. Interestingly, our comprehensive proteomic profiling of pre-diagnostic plasma collected from patients who were diagnosed with lung cancer within two years revealed that circulating TGF-β levels were associated with the timing of the blood draw and levels of a set of circulating proteins known to be immunogenic in lung cancer. Our data suggested that, with the increase of TGF-β and the switch of its functions, the levels of tumor antigens increased, but the levels of autoantibodies against tumor antigens decreased (unpublished data). Those findings suggest that circulating TGF-β levels are associated with not only tumor biology but also systemic host response to tumors, which precedes clinical diagnosis, and thus that TGF-β, antigens emanating from tumors, and autoantibodies against tumor antigens are potential biomarkers for early detection of cancer.

## 5. Conclusions and Perspectives

The strategy of targeting TGF-β has been investigated in many different cancer types. However, TGF-β has different functions during cancer initiation and development, which may cause a failure of therapy targeted to TGF-β signaling. The switching roles of TGF-β have been studied for decades, and much effort has gone into the development of this targeted therapy. Recently, a few important proteins have been identified as the key switches, most of which were verified only in particular types of cancer. There is an urgent need to develop novel therapeutic strategies to address TGF-β signaling and to discover new biomarkers to optimize the timing of the therapy, which together could significantly impact cancer patient care in the new era of personalized cancer medicine [99].
